# Supplementary material for: Are carbon emissions trading and green financial instruments synergistic? -Comprehensive quantitative research based on content analysis
Source: PLoS One. 2024 Mar 7;19(3):e0298601. doi: 10.1371/journal.pone.0298601 (PMC10919601; doi:10.1371/journal.pone.0298601)
Supplement: S1 Data — (ZIP) [file pone.0298601.s001.zip › Data/地级市Z_F工作报告.zip!/╨┬╜«/╬┌┬│─╛╞δ╩╨/└┤╫╘╣1⁄2╓┌║┼í╛┬φ┐╦╩2╛▌═°í┐_2018.docx]

各位代表：

现在，我代表市十六届人民政府，向大会报告工作，请予审议，并请各位政协委员、列席代表提出意见。

2017年工作回顾

刚刚过去的一年，是全市上下向着更高水平小康社会奋力迈进，不断开创现代化国际城市新局面的关键一年。一年来，在自治区党委、自治区人民政府和市委的坚强领导下，在市人大、市政协的监督支持下，我们以迎接党的十九大、学习宣传贯彻党的十九大精神为主要任务，深入贯彻以习近平同志为核心的党中央治疆方略，紧紧围绕社会稳定和长治久安总目标，全面落实自治区党委、自治区人民政府和市委的决策部署，坚持稳中求进工作总基调，积极适应经济发展新常态，贯彻新发展理念，全力抓好反恐维稳、经济发展、城市建设“三件大事”，首府各项事业取得显著成绩，圆满完成市十六届人大一次会议确定的各项目标任务。特别是通过不懈努力成功创建全国文明城市，城市文明达到了一个新高度，首府有了一个含金量最高、综合性最强、影响力最大的城市品牌，为全面建成更高水平小康社会奠定了坚实基础。

一、社会稳定基础更加巩固

（一）严打专项行动不断深入。坚决落实自治区党委反恐维稳“组合拳”，坚持严打力度不减、势头不变、措施不松，对暴 恐分子形成了强大震慑。

（二）社 会 面 防控不断强化。构筑起以便民警务站为依托的社 会 面防控网，以社会单位尤其是学校、医院、商场等人员密集场所为主的内部自保网，居民小区封闭式管理网。

（三）基层基础不断夯实。积极推行社区阵地标准化建设，落实考核奖惩和激励保障机制，不断激发社区工作活力。扎实开展“访惠聚”活动，全力抓好维护稳定和服务群众工作，不断筑牢维护稳定的基层基础。

（四）民族团结宗教和睦不断加强。大力开展马克思主义“五观”“三个离不开”和“五个认同”宣传教育，不断筑牢中华民族共同体意识。认真开展“民族团结一家亲”活动，各族干部职工和基层群众互相结对认亲，促进了各族干部群众交往交流交融。依法加强宗教事务管理，积极引导宗教与社会主义社会相适应。

二、经济持续健康快速发展

预计全年实现地区生产总值2700亿元，增长8%；完成全社会固定资产投资2020亿元，增长25.7%；社会消费品零售总额1317亿元，增长6.5%；实现全口径外贸进出口总额917亿元，增长5%；一般公共预算收入400.7亿元，增长8.4%，突破400亿元大关；城镇居民人均可支配收入36993元，增长8.2%；农村居民人均可支配收入17790元，增长9%；居民消费价格上涨3%。

（一）固定资产投资规模创历史新高。我们始终坚持把投资作为拉动经济增长的主要动力，围绕补短板、添动能，突出现代化国际城市和丝绸之路经济带核心区“五大中心”建设，大力开展精准招商，谋划做实重点项目库，以服务强度换落地速度，形成“储备一批、建设一批、竣工一批”的良性循环。我们出台了一批振兴实体经济、拓宽融资渠道、激发社会领域投资活力的政策措施，积极开展集中联审联批，探索了一条抓项目、促投资的新路子，确保了老城区改造提升、轨道交通等651项重点项目顺利实施，固定资产投资首次跨越2000亿元大关，创历年最好。

（二）“三去一降一补”持续深入。围绕供给侧结构性改革，抓好五大任务落实，化解钢铁落后产能180万吨，关停煤矿17家、去产能148万吨；采取货币化安置等措施，有效推进房地产去库存；全面落实国家、自治区各项减免税政策，清理规范涉企收费38项，惠及企业8.5万家，累计减轻企业负担17.3亿元。行政审批、国资国企、财税金融、生态环保等领域薄弱环节不断加强，有效弥补了制约经济社会发展的短板。

（三）新型工业化步伐加快。实现工业增加值630亿元，增长10%。产业集群发展稳步推进，装备制造、新能源、新材料等产业已培育骨干企业40余家、相关配套企业80余家。工业绿色化发展迈出坚实步伐，经济技术开发区（头屯河区）获批国家绿色园区，金风科技获批国家绿色供应链示范企业，新特能源、新疆众和、阜丰生物3家企业获批国家绿色工厂。产品技术水平明显提升，数字化抽油机达到国际领先水平，2.5兆瓦风力发电机组、光伏逆变器、高压玻璃钢油管等产品达到国内领先水平。“丝绸之路号”地铁列车成功下线，填补了新疆轨道交通装备制造业空白，广汽自主品牌新疆项目主体完工。获批全国产融合作试点城市，产业、金融创新发展优势显著增强。

（四）现代服务业持续发展。软件信息、电子商务等新兴业态加速集聚，新增国家级电子商务示范企业1家，新疆软件园绿谷创新中心正式运营。城市共同配送网络体系不断完善，发展配送点1000余家，城市连锁便利店增长30%以上。特色餐饮集群化、品牌化发展取得新进展，新增餐饮钻级酒家28家、餐饮名店22家、餐饮老字号11家。成功举办第七届乌鲁木齐国际食品餐饮博览会等123个展会，签约1644亿元。丝绸之路经济带旅游集散中心试运营，丝绸之路国际度假区被认定为“国家体育旅游示范基地”。全年接待国内外游客3300万人次，实现旅游总收入450亿元，均增长30%。

（五）农业农村工作再上新水平。农业种植结构不断优化，高附加值农产品种植面积稳步增加，完成设施农业提质增效3000亩。高标准基本农田建设稳步推进，完成全域永久基本农田划定工作。农产品风险监测合格率达99.3%，农产品质量安全监管水平不断提高。全面启动水西沟“冰雪特色小镇”、安宁渠“记忆休闲小镇”、达坂城“王洛宾情意小镇”建设。新建农村公路100公里，实施饮水安全巩固提升项目3个，乌鲁木齐县集镇区实现清洁能源供暖全覆盖。

（六）科技支撑作用显著增强。“双创示范”城市建设加快推进，新认定国家级高新技术企业32家、自治区级“专精特新”企业17家，新增自治区级企业技术中心4家、市级创新型（试点）企业58家。设立生态环境与污染治理、轨道交通等重大科技专项，建立小微企业创业创新先导区9个，新建众创空间等新型孵化载体60个。鼓励和保护知识产权，全年专利申请5600余件。顺利通过国家创新型试点城市建设验收，经济技术开发区（头屯河区）获批全疆首家国家知识产权示范园区，高新技术产业开发区（新市区）获批国务院第二批大众创业万众创新示范基地。

面对稳定发展两个“三期叠加”严峻形势，全市固定资产投资、财政收入实现新突破，经济实现持续快速健康发展，实属不易。这是全市各族干部群众用辛勤汗水换来的，用不服输的斗志、敢担当的魄力拼出来的，用改革创新、连续作战的精神奋斗出来的。这再次证明，任何艰难险阻都阻挡不了我们昂首前行的步伐！

三、城市建设日新月异

我们围绕打造现代化国际城市，以接受中央环保督察为契机，坚持高标准规划、高水平建设、高质量管理、高优质服务，着力推进“树上山、水进城、地变绿、煤变气、天变蓝、城变美”六项重点工作，加强生态修复和城市修补，我市被列为全国第三批生态修复、城市修补试点城市，人居环境和城市品质全面提升，美丽首府建设迈出坚实步伐。

（一）城市规划建设管理水平不断提升。高标准启动城市总体规划（2016—2035年）修编，被国家住建部确定为城市总体规划编制试点城市。深入推进主体功能区规划实施，调整完善土地利用总体规划。高起点编制老城区改造提升规划，促进老城提品位。突出抓好城市新区、临空经济示范区、城南经贸合作区规划，促进新区出形象。着力完善国际陆港区、南山国际旅游区、水磨沟区工业园、甘泉堡经济技术开发区、特色金融服务区规划，促进特色产业集聚发展。智慧城市建设加快推进，组建大数据局，优化整合数字化城市管理平台。制定城市管理新标准，完善环境综合治理四级网络管理模式。城区生活垃圾无害化处理率达98.7%。无线城市建设取得新进展，布设无线接入点2.1万个，全面建成“光网城市”。

（二）城市基础设施建设日趋完善。实施克南高架东延二期、米东大道二期等61项重要路网工程。轨道交通1号线北段试通车，2、3、4号线建设有序推进。建成苏州路西延、艾丁湖路综合管廊。完成老旧供热管网改造178公里，新建燃气管网226公里。大黑沟固废综合处理厂建设，河西、七道湾污水处理厂提标改造，乌鲁木齐河防洪工程加快推进。建成装配式建筑产业基地6家，全疆首个装配式项目东进场路高架启动建设，完成装配式建筑100万平方米。

（三）老城区改造提升力度空前。投入870亿元，完成21.7万余户、1730余万平方米老城区改造征收任务，29个重点改造片区征收工作已全部完成，征收量为历年棚户区改造征收总和的3.6倍。改造提升市政管网90公里、道路20公里。实施珠江路东延、新市路片区路网完善等工程，有效改善了老城区交通环境。棚户区改造扎实推进，新开工房屋6170套，面积近71万平方米，复工房屋2.5万套，面积392万平方米。老城区特色更加鲜明、公共服务设施更加完善，赋予了老城区新功能、新活力，探索出一条加快老城区转型升级的新路径。

（四）城市靓化亮点纷呈。投入60亿元，大力实施以建筑节能保温为主的靓化工程，完成50条街区、1721栋建筑外立面改造和靓化提升，铺设道路82万平方米，线缆入地75公里。强化杆线整治、门头牌匾、户外广告、公共厕所、垃圾运输、环境卫生治理，拆除违法建筑242万平方米。建设路靓化示范街等城市街区旧貌换新颜，城市形象更加靓丽，让各族群众切身感受到了经济社会发展给首府城市面貌带来的巨大变化，获得感、幸福感、自豪感显著提升。

（五）城市生态绿化建设加快推进。新增绿地1.8万亩、花园式单位20个。完成森林抚育1万亩、大苗基地建设1100亩、退耕还林6000亩、裸露荒山绿化6129亩。新建小游园及街旁绿地101块、小绿道9条，启动大湾片区棚户区、东大梁片区二道湾公园绿化景观改造提升工程。十七户湿地公园、和平渠大寨闸公园“水进城”先导工程加快推进，柴窝堡国家湿地公园正式挂牌。国家生态园林城市创建工作取得新成效。

（六）大气污染治理深入推进。强化“控煤、控尘、控污、控车”协同治理，强力推进“煤变气”工程，拆改燃煤供热设施3.2万台、工业锅炉393台。淘汰“黄标车”1.5万余辆，升级改造建筑垃圾运输车1150辆。完成7家电厂15台机组超低排放改造，整治“散乱污”企业4121家，关停2294家。实施高铁片区新能源供热示范项目，新增风电采暖装机85兆瓦，完成既有建筑节能改造80万平方米。空气质量持续改善，细颗粒物（PM2.5）和可吸入颗粒物（PM10）平均浓度较同期分别下降6.6%和8.6%。

（七）城市生态修复取得新进展。加强水源地综合整治，完善河长制组织体系，严格落实水资源管理“三条红线”，严格执行饮用水保护和取水许可制度。落实水污染总量减排，集中治理工业集聚区废水。编制土壤污染治理与修复规划，推进矿产资源开发治理与修复，关闭非法砂场、碎石场107家，私挖滥采行为得到有效遏制。启动环鹏公司后峡工业生产基地搬迁工作，一号冰川、柴窝堡湖等重点水源涵养区生态修复治理不断加强。

实践告诉我们，只要我们始终坚持在保护中有序发展、在发展中有效保护，切实把发展建立在生态环境安全的基础上，就一定会建成天蓝地绿水清的美丽首府！

四、民生改善取得新成效

（一）安全惠民再提升。平安首府建设深入开展，“平安细胞”工程建设扎实推进。国家食品安全示范城市创建取得阶段性成效，食品药品监管不断强化。粮食安全工作持续加强。电梯应急处置服务平台建设加快推进，1.5万部电梯实行电子监管。车用气瓶电子标签系统实现封闭运行。安全生产形势持续稳定好转，应急管理能力和自然灾害综合保障能力逐步增强，各类突发事件和自然灾害得到有效应对和处置。

（二）就业惠民再加强。城镇新增就业11.9万人，转移农村富余劳动力7371人次，城镇登记失业率为3.03%。培育创业孵化基地6家，扶持创业企业2413家，带动就业3.5万人。发放各类就业补贴和小额担保贷款4.5亿元，惠及劳动者6.8万人。开展职业培训11.9万人。全市最低工资标准提高到每月1620元，为1.26万名农民工解决拖欠工资1.86亿元。

（三）教育惠民再深入。全面实行农村3年、城镇2年学前免费教育，统一城乡义务教育“两免一补”政策，实施普通高中和中等职业教育免学费政策，免除各类教育费用8.9亿元，惠及近37万名学生。投入3.44亿元新建农村幼儿园42所，1.69万农村适龄幼儿实现学前免费教育。全面普及和推广国家通用语言文字教学，巩固义务教育均衡发展成果，推动优质教育资源北扩和普通高中扩容提质，市第1中学新校区全面投入使用，市第130中学、15小学北校区等10所新建中小学招生办学。推进职业教育现代化，促进人才培养与岗位需求有效衔接。

（四）医疗惠民再强化。实施全民健康体检工程，实现常住人口全覆盖。医药卫生体制改革加速推进，公立医院以药补医格局彻底打破。新型医联体、分级诊疗模式初步形成，市属医院与北京、上海等25家知名医院、疆内114家医院建立专科联盟。投入5116万元建成一批基层医疗服务设施，实现乡镇卫生院、村卫生室标准化建设全覆盖。建成全民健康云数据中心，实现区域内医疗机构数据共享。取消三级医疗机构定点选择限制，基本用药目录增加药品2500余种，让群众就医看病更方便。艾滋病、结核病等重大传染病防控取得新进展。中医民族医药事业健康发展。人口和计划生育服务管理不断加强，两孩政策平稳实施。

（五）社保惠民再提高。连续13年提高企业退休人员养老金，人均每月达3014元，惠及26万人。城乡居民基础性养老金标准增加至每人每月180元，3.68万城乡居民受益。全面实施城镇职工大病保险制度、门诊统筹制度，减轻群众经济负担9000余万元。与31个省、自治区、直辖市医疗机构实现异地就医直接结算。落实工伤保险费率动态调整机制。深入推进全民参保计划，超额完成养老、医疗、工伤、失业、生育保险扩面提标任务。

（六）扶贫惠民再夯实。城市低保和农村低保标准分别提高到每人每月450元和280元，城市低保对象中“三无”人员全部纳入供养特困人员范围。发放救助金1.7亿元，救助困难人员10.5万人次，向困难学生发放营养膳食补贴238万元，兜住兜牢困难群众生活底线。开展疆内对口协作扶贫，实施产业、教育、医疗和就业精准帮扶。

（七）安居惠民再升级。投入6亿元，完善“两居”工程配套基础设施。筹集专项资金1.28亿元，建设安居富民房5003户。将住房保障申请家庭人均月收入标准调整到2850元，住房保障覆盖面进一步扩大。完善和创新保障性住房摇号分配方式，解决9000余户中低收入家庭住房问题，实现保障房分配常态化。

（八）暖心惠民再持续。新设立许可养老机构2个，新建农村幸福互助院9个、社区老年人日间照料中心6个。为4.5万余名80周岁以上老年人发放高龄津贴3105万元。深入开展双拥共建活动，落实退役士兵创业扶持政策，发放各类慰问、优抚金1.2亿元。办理法律援助案件2619件。新建立体停车库15处，鼓励企事业单位面向社会提供错峰停车服务，进一步缓解停车难问题。坚持“公交优先”发展战略，更新公交车404辆，优化调整公交线路15条，新增出租车700辆。投放政府储备肉3200吨、蔬菜5万吨，居民基本生活品价格保持稳定。

（九）文化惠民再深化。投入5000万元，扶持奖励优秀文化体育产业项目。发行文化惠民卡1万张，举办惠民演出300余场，放映公益电影1.1万场。新增市级文化产业示范基地21家、特色文化街区2条、文化产业示范园区4个、国家体育产业示范基地1家。为212个社区配备公共体育设施，举办首届戏曲艺术节和市第四届运动会。成功创建国家文化消费试点城市。新闻出版、质监、气象、地震、地方志、档案等工作取得新进展，妇女儿童、残疾人和红十字事业取得新成绩。

“九大惠民工程”的生动实践告诉我们：只要永远把人民对美好生活的向往作为奋斗目标，永远与人民同呼吸、共命运、心连心，多谋民生之利、多解民生之忧，就一定会不断凝共识、汇众智、聚众力，凝聚起实现总目标的磅礴力量！

五、改革开放实现新突破

（一）重点领域改革全面深化。“放管服”改革力度不断加大，“一门式、一网式”政务服务模式和“互联网+政务服务”加快推进，“两集中、两到位”改革稳步实施，“减证便民”专项行动扎实推进。房地产交易实现一站式办理。深化商事制度改革，全面实施企业登记“多证合一、一照一码”，各类市场主体增长12.3%。大力推广政府与社会资本合作模式，落地项目30个，总投资1470亿元。国资国企改革持续推进，国有企业产权多元化、资本证券化取得新成效，完成新疆城建重大资产重组。成立城市建设发展基金和产业引导基金，市级国有小额贷款公司设立工作加快推进，国有融资性担保公司资本实力得到提升。企业上市取得新进展，3家企业成功上市。深化城市管理体制改革，完成供热、燃气等22项管理职能划转，大城管格局初步形成。

（二）丝绸之路经济带核心区“五大中心”建设加快推进。我们坚持把抓好丝绸之路经济带核心区“五大中心”建设，作为扩大对外开放的重中之重。交通枢纽中心辐射带动作用显著增强。乌鲁木齐国际机场改扩建工程加速推进，航空、铁路、公路等重大基础设施不断完善，内外联通“动脉”进一步畅通。商贸物流中心配套服务能力显著提升。全国首趟集拼集运中欧班列和全疆首列铁海联运班列成功开行，中欧、中亚、中俄班列全年开行600余列。综合保税区运行机制不断完善，丝绸之路经济带创新驱动发展试验区建设加快推进。启动跨境电商综合试验区申报工作，启用乌鲁木齐国际机场跨境电子商务分拣清关中心，机场进口冰鲜水产品指定口岸通过国家验收。国际贸易服务区一期主体完工，已注册企业23家。跨境电子商务公共服务平台上线运营。金融服务中心市场主体日趋多元。启动绿谷金融港建设，中亚五国金融投资服务中心加快推进。跨境贸易与投资人民币结算试点工作有序开展，新疆黄金交易中心即将组建成立。文化科教中心基础进一步夯实。市文化中心“六馆”、奥林匹克体育中心等一批公共文化体育项目扎实推进。医疗服务中心集聚效应初见成效。新疆国际健康产业园、国际医疗综合体项目相继启动。成功举办丝绸之路国际健康产业博览会及各类“丝绸之路健康论坛”34个。跨境远程医疗服务平台已连接3个国家24家医院，跨境医疗旅游推介平台辐射18个国家，全年接待外籍患者近4000人次。

六、政府自身建设全面加强

我们把坚持党的领导作为做好政府工作的根本保证，牢固树立全心全意为人民服务的宗旨，坚持依法履行政府职责，确保党中央、自治区党委和市委的决策部署得到不折不扣的贯彻落实。自觉接受市人大及其常委会的依法监督和市政协的民主监督，积极听取各民主党派、工商联、无党派人士和人民团体意见，办理自治区、市人大代表议案、建议和政协提案395件，办复率100%。政务公开工作步入制度化、规范化轨道，相关工作经验在全疆推广，政府工作透明度不断提升。“两学一做”学习教育常态化制度化，“学、转、促”专项活动扎实开展，持续向“三 股 势 力”“两 面 人”发声亮剑。持之以恒贯彻落实中央八项规定、自治区党委和市委十条规定，坚决纠正“四风”“四气”，持续开展作风建设“三项治理”，干部作风不断改善。依法治市工作持续深化，信访和行政调解工作机制进一步完善。行政监察、审计监督工作扎实推进，严肃查处违纪违法案件，反腐倡廉取得新成效。

各位代表，回顾一年来的工作，成绩来之不易。我们深深感到：在社会稳定的基础扎牢之后，首府经济发展活力、动力得到了进一步激发，市民的安全感、幸福感、自豪感也得到了大幅提升。所有这一切，是以习近平同志为核心的党中央治疆方略英明引领的结果，是自治区党委、自治区人民政府和市委正确领导的结果，是全市各族人民共同努力和各方面大力帮助支持的结果。在此，我代表市人民政府，向全市广大工人、农民、干部和知识分子、兵团职工，向各民主党派、无党派人士，向人民团体和社会各界人士，向驻乌人民解放军和武警部队指战员、公安干警，向中央驻乌单位、所有援乌干部，向人大代表、政协委员表示衷心的感谢并致以崇高的敬意！

在肯定成绩的同时，我们也清醒地看到工作中还存在着问题和不足：一是新疆处于暴 力恐怖活跃期、反分裂斗争激烈期、干预治疗阵痛期“三期叠加”的态势没有根本改变，落实总目标的任务依然艰巨而繁重。二是发展不平衡不充分的一些突出问题尚未解决，发展质量和效益还不高，构建现代化经济体系，实现提质与增量同步发展还任重道远。三是实体经济支撑力不足，特别是高新技术产业支撑作用不明显，新产业新业态尚未形成规模，产业转型升级缓慢。四是基础设施建设相对滞后，城市规划建设管理与国际化水准尚有较大差距。五是基本公共服务优质化、均等化程度不高，就业、教育、医疗和社会保障等领域还有不少短板，住房、环境保护等方面工作还需进一步加强完善。六是一些干部作风漂浮、政策棚架，“四风”“四气”还不同程度存在。面对这些问题，我们必须迎难而上、敢于担当，以创新的思维、必胜的勇气，下大力气来解决。

2018年主要工作

2018年，是我们全面贯彻党的十九大精神的开局之年，是改革开放40周年，是决胜全面建成更高水平小康社会、实施“十三五”规划承上启下的关键一年，也是我们巩固社会稳定和长治久安新胜利的重要一年。

2018年全市工作总体要求是：高举中国特色社会主义伟大旗帜，深入学习贯彻党的十九大精神，以习近平新时代中国特色社会主义思想为指导，认真学习贯彻习近平新时代中国特色社会主义经济思想，贯彻落实以习近平同志为核心的党中央治疆方略，牢固树立社会稳定和长治久安总目标，全面落实自治区党委经济工作会议各项部署，坚定市委确定的奋斗目标和工作思路不动摇，打好反恐维稳“组合拳”，坚持稳中求进工作总基调，坚持新发展理念，紧扣社会主要矛盾变化，按照高质量发展的要求，统筹推进“五位一体”总体布局和协调推进“四个全面”战略布局，坚持以供给侧结构性改革为主线，统筹推进稳增长、促改革、调结构、惠民生、防风险各项工作，推动质量变革、效率变革、动力变革，奋力开创和谐稳定新局面，奋力开创改革发展新局面，奋力开创民生改善新局面，奋力开创民族团结新局面，奋力开创宗教和谐新局面，奋力开创意识形态领域工作新局面，奋力开创生态良好新局面，努力建设团结和谐、繁荣富裕、文明进步、安居乐业的中国特色社会主义美丽首府。

2018年全市经济社会发展主要预期目标是：地区生产总值增长7.5%，固定资产投资增长15%，社会消费品零售总额增长7.5%，力争外贸进出口总额增长15%，一般公共预算收入增长7.5%，城镇居民人均可支配收入增长9%，农村居民人均可支配收入增长10%，城镇登记失业率控制在3.7%以内，居民消费价格涨幅控制在4%左右。

我们深深感到，实现上述目标，必须积极适应新时代、聚焦新要求、落实新部署，坚持稳中求进工作总基调，坚持贯彻落实新发展理念，坚持以供给侧结构性改革为主线，坚持以创新引领发展，坚持以人民为中心的发展思想，坚持以总目标为统领，以钉钉子精神做实做细做好各项工作。

一、全力确保社会大局持续稳定

（一）推动反恐维稳工作常态化。强化“打防管建”各项措施落实，在任何时候都要保持对暴 恐分子的严打高压态势。坚持专门机关和发动群众相结合，健全完善协调联动机制，构建无缝隙、无盲区、无空白的群防群治格局。

（二）加强基层治理体系和治理能力建设。以提升基层社会治理社会化、法治化、智能化、专业化水平为着力点，增强管委会（街道、乡镇）、社区（村）统筹辖区社 会 面 防控能力，将各类维稳力量紧紧团结凝聚起来，实现对区域内人、事、物的统一管理，做到协调联动、一体互动，形成强大反恐维稳合力。

（三）扎实做好意识形态领域反分裂斗争。深入开展马克思主义“五观”“五个认同”和新疆历史、民族发展史、宗教演变史宣传教育，引导各族群众牢固树立国家意识、公民意识、中华民族共同体意识。持续深入开展发声亮剑活动，弘扬主旋律、传播正能量。扎实推进“去极端化”工作，确保意识形态领域安全。

（四）广泛宣传动员各族群众。深入做好“宣传群众、帮助群众、组织群众”三件事。深入推进“访惠聚”工作，大力开展党的十九大精神和国家政策法律知识宣传，不断增强各族群众的法律意识和社会主义道德观念。大力开展群众帮扶工作，让各族群众深切感受到党和政府的温暖。广泛动员组织基层群众，形成打防暴 恐活动的强大合力。

二、促进实体经济转型升级

党的十九大作出了“贯彻新发展理念，建设现代化经济体系”的战略部署，这是实现更高质量、更有效率、更加公平、更可持续发展的必由之路。我们要按照“一产上水平、二产抓重点、三产大发展”的要求，以供给侧结构性改革为主线，进一步加大招商引资力度，加快构建“大企业顶天立地、小企业铺天盖地”的产业格局，奋力开创经济发展新局面。

（一）坚定不移走新型工业化道路。围绕化工产业集群，重点打造米东区精细化工小微企业园、甘泉堡化工产业配套园，加快推进华泰重工绿色生产技术和应用项目建设。支持化工产业技术改造升级，依托石油资源优势和现有产业基础，发展石油下游产业和精细化工产品，加快打造石油化工集群、绿色煤化工集群和先进氯碱集群。围绕先进装备制造产业集群，打造经济技术开发区（头屯河区）装备制造配套产业园，重点培育风电装备、汽车及零部件、轨道交通、农业机械、纺织机械和高性能钢铁产品等产业链，大力推进卓郎智能纺机装备制造等项目建设，促进工业技术改造，推动制造业向价值链中高端延伸。围绕新材料产业集群，打造甘泉堡新材料产业园、达坂城区新型建筑产业园和建筑新材料产业园，做大做强硅（铝）基新材料、高分子材料等产业，培育新材料产业基地。围绕智慧安防产业集群，打造高新技术产业开发区（新市区）智慧安防产业园，引进国际先进技术企业，成立国内最大的智慧安防产业联盟，积极搭建社会安全风险感知与防控国家实验室、国家级超算中心、中国特种汽车研究院等国家级研发平台，全力打造具有创新活力和全球市场竞争力的智慧安防产业。围绕轻工制品产业集群和纺织产业集群，打造经济技术开发区（头屯河区）消费品工业小微企业园，积极推进水磨沟区工业园建设。加快乌鲁木齐国际纺织品服装商贸中心建设，构建纺织服装产品交易和集散平台。

（二）促进现代服务业实现大发展。加快构建差异化、特色化、便利化的现代商贸服务体系，优化提升商贸服务业，重点打造沙依巴克区商贸服务产业园。加快推进电子商务和跨境电商发展，在综合保税区打造全疆首个跨境电商公共服务中心。围绕特色餐饮集群建设，加快特色餐饮企业转型，构建满足多样化消费需求的特色餐饮服务体系。围绕商贸物流产业集群，重点打造国际商贸物流产业园区、甘泉堡工业物流园、天山区城市物流产业园。以国际陆港区、临空经济示范区为重点，提升中欧班列、多式联运、国际航空物流综合配套服务能力。加快现代金融服务体系建设，积极引入各类金融机构，加快绿谷金融港项目建设，推动重大金融产业项目实施。做大做强旅游业，积极开展全域旅游示范市建设，加快推进南山国际旅游区建设，打造旅游集散服务综合体，做优精品线路，打造特色品牌，力争将首府建成国际知名的运动休闲度假旅游目的地和国家级生态旅游示范区。大力发展信息服务业，加快新疆软件园云计算基地建设，推进新疆深圳信息合作示范园、物联网科技产业园、新疆工业云和大数据创新中心、丝路声谷等项目落地。办好第六届中国—亚欧博览会和第五届丝绸之路经济带城市合作发展论坛。支持健康服务业发展，加快国际健康产业园和全疆首家医养结合综合服务园等项目建设，推动医养一体化快速发展。

（三）促进农村农业发展上水平。把握农村发展规律，顺应城乡融合发展大趋势，坚持新型城镇化和乡村振兴两手抓，充分发挥新型工业化、信息化、城镇化对乡村振兴的辐射带动作用，积极推动基础设施向农村延伸、公共服务向农村拓展、产业向农村合理布局。以服务城市、满足城乡居民生活需求为重点，扎实推进种植业、养殖业结构调整，大力发展现代畜牧业，壮大特色农业和设施农业，推动农业高质量发展，不断增加绿色优质农产品供给。以推进农业产业化经营、发展农副产品精深加工为主攻方向，发展和引进一批带动力强的农副产品深加工龙头企业，促进农村一二三产融合发展，促进农牧民增收。

（四）强化科技创新驱动。强力推进乌昌石国家自主创新示范区、国家创新型城市和“双创示范”城市建设。围绕先进装备制造、新能源、新材料、现代服务、公共安全等领域，以国际科技合作中心为核心，推进新疆高端人才创新创业大厦、科技领军人才创新驱动中心等基地建设，打造高新技术产业开发区（新市区）创新创意小微产业园、环保产业园，加快推进检验检测认证产业园建设，营造良好创新创业环境。促进科技与金融深度融合，实施“旅游+科技”发展战略。加强工程技术研究中心、重点实验室建设，培育高新技术企业、创新型（试点）企业60家，企业技术中心、产学研联合开发示范基地6家，力争新增“专精特新”企业15家。进一步加大知识产权保护力度，保持专利申请量持续稳步增长。

三、加快完善现代化国际城市功能

（一）强化规划的引领调控。以新发展理念统筹推进城市规划编制试点工作，修编完善主体功能区规划，科学划定市域“三区三线”空间格局，确定开发强度和管控措施，破解城乡二元结构，提升生态系统治理能力，促进区域协调发展。积极推动“多规合一”，调整优化重大产业、区域交通布局和生态环境保护区域，形成人口、经济、资源环境合理布局的空间开发格局。依法加强城市规划管理，坚决维护规划的严肃性。

（二）高水平推进城市建设。完善城市“三环十五射”路网骨架，增强首府重点区域和新区基础设施配套能力，科学有序拓展城市发展空间。实施华光街东延、东进场高架等道路建设，建成克南高架二期项目。新建西二环快速路、绕城高速东线观园路立交至达坂城公路，完成南山旅游公路主线工程。优化市域路网，打通丁字路、断头路50条，续建地下综合管廊50公里。轨道交通1号线建成并投入运营，加快2、3、4号线建设。建成空港长途汽车综合客运枢纽。

（三）高质量推进城市精细化管理。以提高城市管理执法数字化、网络化、智慧化水平为目标，深入推进智慧城市建设，完善数字化城市管理平台功能，让城市管理更智能。以推进“厕所革命”为重点，完善市政设施功能，让城市服务更便利。加强城市主干道沿线环境整治，积极推进环卫作业服务市场化，加快大黑沟生活垃圾焚烧发电项目建设，持续推进背街小巷、老旧小区、城乡结合部环境卫生治理，让市容市貌更洁净。进一步明确城市管理和执法职责边界，把严格规范公正文明执法要求，落实到城市管理执法全过程，让城市管理更文明。

四、加快推进美丽首府建设

党的十九大报告指出：“必须树立和践行绿水青山就是金山银山的理念。”我们要坚持新发展理念，加快推进“树上山、水进城、地变绿、煤变气、天变蓝、城变美”六项重点工作，推进生态修复、城市修补工作，坚决打好污染防治攻坚战，努力让首府天更蓝、地更绿、水更清、城更美，不断满足各族人民日益增长的优美生态环境需要。

（一）大力推进“树上山”，打造城市绿肺。全力推进雅玛里克山等9个裸露荒山绿化项目，完成绿化总面积约1.5万亩。继续推进荒山绿化提升改造公园建设，完成平顶山公园绿化工作，通过山体绿化及基础设施完善、服务设施建设，完善公园游憩功能，改善首府生态环境和市民休闲环境。

（二）大力推进“水进城”，打造城市水景。合理利用再生水和生态湿地资源，实施再生水北水南调，沟通主要河湖水系，打造水绿融合的复合型生态廊道。按照6条主水系、7条支水系格局，推进“三河、五带、十湖、多点”水生态景观建设，建成河湖水系连通工程一期、大寨闸和十七户湿地先导工程，整治、新挖河道125公里，新建水景观865公顷。初步实现200米见绿、500米亲水、5千米畔河、1万米见湖。

（三）大力推进“地变绿”，打造城市绿脉。按照“宜树则树、宜花则花、宜草则草”的原则，大力开展街道社区、背街小巷、居民小区等绿化美化工作。新增、改造绿地1.3万亩，新建小游园100个、小绿道8条，创建花园式单位20个。完成森林抚育1万亩，建设大苗基地2900亩。加快东绕城生态圈绿化工程建设，加大公园绿地建设改造力度，建成区内绿化覆盖率提高到41%以上，绿地率提高到38%以上，人均公园绿地面积增至11.5平方米以上。让百姓“开窗见景、出门见绿”，打造“接触到绿、享受到荫、观赏到景”的宜居环境。

（四）大力推进“煤变气”，打造电气化首府。积极推进电采暖供热工程，着力提高公共机构及民用领域电气化水平，优化供热能源结构，持续减少煤炭消耗。加快实施50吨及以下工业燃煤锅炉电气化改造，在交通运输和旅游领域积极开展电能替代工作。完成燃煤小锅炉电气化改造，拆改城乡结合部自采暖燃煤供热设施1.9万台，逐步实现城市周边区域清洁能源全覆盖。

（五）大力推进“天变蓝”，打好蓝天保卫战。严格落实“控煤、控尘、控污、控车”措施，提高能源使用效率，强化资源环保准入约束。加快园区环保设施建设，推进园区淘汰落后产能。完善石化等重点行业挥发性有机物监测监控体系建设。加大施工、道路、运输扬尘的防治力度。加强散煤和“散乱污”企业清理整治。加大充电基础设施建设，积极推广新能源汽车应用。继续实施机动车尾气排放增量控制，强化重污染天气应对措施，严格落实区域联防联控，完成10万千瓦及以上燃煤发电机组超低排放改造。加快达坂城区清洁能源规模化示范基地建设，积极推进清洁能源规模化循环利用，为打赢蓝天保卫战奠定坚实基础。

（六）大力推进“城变美”，打造魅力城区。按照“两年见效”目标，全力推进老城区改造提升，完成9.5万户征收任务，积极构建“6中心、6园”多中心、组团式发展格局。进一步加强配套市政基础设施建设，完善公共服务配套，增加绿地空间，确保老城区改造提升工作取得阶段性成效。加快推进以建筑节能保温为主的靓化工程，进行以建筑屋顶隔热、防水、防凌为主的“平改坡”改造，力争完成主城区所有街道靓化任务，实现街道清新亮丽、环境洁净有序。全面建成水西沟“冰雪特色小镇”、安宁渠“记忆休闲小镇”、达坂城“王洛宾情意小镇”，年底前实现开门迎客。大力开展“城市病”环境综合整治，提升城市整体环境品质。我们就是要通过一条街道接着一条街道地整治提升，一茬压着一茬地干，努力在建设美丽首府上取得新突破，实现城乡面貌焕然一新。

五、奋力开创民生改善新局面

党的十九大报告指出：“增进民生福祉必须多谋民生之利、多解民生之忧。”我们要始终把首府各族人民利益摆在至高无上的地位，通过持续推进“九大惠民工程”，让改革发展成果更多更公平惠及首府各族市民，使人民获得感、幸福感、安全感更加充实、更有保障、更可持续。

（一）继续实施安全惠民工程。深化安全生产领域改革，狠抓重点行业领域安全监管，保障电梯等重点特种设备使用安全，坚决防范遏制重特大安全生产事故。大力实施食品安全战略，加强食品药品监管，确保人民群众饮食用药安全。加强洪涝、地质灾害等防灾减灾工作，完善监测预警、救援救助机制，提升突发事件应急处置能力和保障水平，确保人民群众生命财产安全。

（二）继续实施就业惠民工程。坚持就业优先战略和积极就业政策，加快公共就业服务体系建设，抓好重点群体就业创业。新增城镇人员就业10万人以上，大中专毕业生就业率达85%以上，农村富余劳动力转移就业6500人次。实施“万人技能培训计划”，完成培训9万人，新增创业人员4000人以上。完善政府、工会、企业共同参与的协商机制，全面构建和谐劳动关系。

（三）继续实施教育惠民工程。围绕办好人民满意的教育，持续加大教育投入力度。积极推进市第20中学、第23中学等新校区建设，不断扩大优质教育资源供给。抓好少数民族国家通用语言文字教育，办好特殊教育，促进民办教育健康发展。深化职业教育改革，提高职业教育的社会服务能力。

（四）继续实施医疗惠民工程。加快推进“健康首府”建设，完善全民健康体检长效机制，科学指导群众健康生活。全面深化医药卫生体制改革，健全现代医院管理制度，完善医联体、分级诊疗制度。加强基层医疗卫生服务体系和全科医生队伍建设，不断提升基层医疗卫生服务能力。加快推进儿童医院北院、国际医疗综合体等项目建设。加强与北京市卫生医疗领域合作，提升重点专科诊疗服务水平。统筹做好人口和计划生育工作。

（五）继续实施社保惠民工程。按照兜底线、织密网、建机制的要求，进一步完善统筹城乡的社会保障体系。认真落实全民参保计划。提高城乡居民基础养老标准，完善城镇职工基本医疗保险、大病保险、门诊统筹制度，实施城镇职工大病保险补充办法，进一步减轻重大疾病患者经济负担。稳步推进医保支付方式改革，基本实现跨省异地就医直接结算。不断健全城乡社会救助体系，继续提高城乡低保标准，积极做好各类专项救助。推进普惠型社会福利体系建设，扎实做好儿童福利和残疾人关爱工作。

（六）继续实施扶贫惠民工程。持续巩固脱贫成果，打好精准脱贫攻坚战。加快推进包村定点对口帮扶、南疆和田地区及克州扶贫惠民工程建设，有组织、成建制、多渠道转移安置南疆富余劳动力。进一步完善医疗救助制度，做好医疗、教育等各类专项救助，实现特困供养人员医疗救助费用全部兜底。

（七）继续实施安居惠民工程。加快建立多主体供应、多渠道保障、租购并举的住房制度，积极发展住房租赁市场。继续实施好农村安居、游牧民定居和城镇保障性安居工程，建设安居富民房1100户，积极推进农村危房改造，切实保障困难家庭住房需求，努力实现“住有所居”。

（八）继续实施暖心惠民工程。鼓励社会资本举办非营利性“医养结合”机构，完善社区养老机构设施和服务功能，满足多层次、多样化的健康养老服务需求。实施早餐惠民工程，建成早（快）餐网点1200个。积极推进慢行网络建设，逐步形成“一主、一辅、一环、十射”慢行网络结构。加快完善公交基础设施功能，优化调整公交线路20条。继续扩大公交优惠换乘范围，逐步覆盖全市180余条公交线路，降低市民出行成本。加快公共停车场建设，严查违章占道停车行为，把人行道还给行人。不断拓展法律援助范围，完善四级便民服务网络。巩固提升全国双拥模范城创建成果，更加注重军民融合，加强军政军民团结。

（九）继续实施文化惠民工程。不断完善文化体育设施，推进文化中心“六馆”、奥林匹克体育中心、京剧团新剧场等项目建设，规划建设城市“十分钟文化圈”。推进文化惠民卡增质扩面，开展惠民文化消费季主题活动。实施传统戏曲文化传承工程，全年举办惠民演出300场。继续实施公益电影放映工程，更好地满足各族群众文化需求。加快促进体育产业发展，广泛开展全民健身活动，构建覆盖全市的全民健身服务网络。推动统计、外事、工商、质监、人防、气象、档案、红十字、新闻出版、史志编纂等各项社会事业加快发展。

六、积极促进民族团结和宗教和谐

党的十九大报告指出：“深化民族团结进步教育，筑牢中华民族共同体意识，加强各民族交往交流交融，促进各民族像石榴籽一样紧紧抱在一起。”“全面贯彻党的宗教工作基本方针，坚持我国宗教的中国化方向，积极引导宗教与社会主义社会相适应。”我们要全面贯彻党的民族政策和宗教工作基本方针，不断开创首府民族团结和宗教和谐新局面。

民族团结是各族人民的生命线。我们要始终高举各民族大团结旗帜，准确把握新形势下民族工作的特点和规律，大力培育和践行社会主义核心价值观，筑牢中华民族共同体意识。深入开展民族团结宣传教育，推进“民族团结一家亲”和民族团结联谊活动制度化、常态化、长效化。扎实开展全国民族团结进步示范市创建，广泛宣传民族团结先进典型事迹，把民族团结教育贯穿到学校教育、家庭教育、社会教育各个环节和各个方面。积极创造条件，推动建立各民族相互嵌入式的社会结构和社区环境，使各族群众学习在一起、生活在一起、工作在一起，促进各民族共同团结奋斗、共同繁荣发展。

全面落实党的宗教工作基本方针，坚持保护合法、制止非法、遏制极端、抵御渗透、打击犯罪的基本原则，依法保障信教群众的正常宗教需求，尊重各族群众习俗，稳步拓宽信教群众正确掌握宗教知识的合法渠道。巩固驻村管寺工作成果，完善“七进两有”工程和“九配备”工作，进一步规范宗教活动服务管理，加强爱国宗教人士培养培训，积极引导宗教与社会主义社会相适应。

七、全面深化重点领域改革和对外开放

党的十九大报告指出：“实现中华民族伟大复兴，必须合乎时代潮流、顺应人民意愿，勇于改革开放，让党和人民事业始终充满奋勇前进的强大动力。”我们要更加自觉地投身改革创新时代潮流，坚持社会主义市场经济改革方向，压茬拓展重点领域和关键环节改革，深度参与国家“一带一路”建设，不断开创首府改革开放新局面。

（一）深化供给侧结构性改革。积极推进要素市场化配置改革，继续推进“三去一降一补”，提高供给体系质量，促进经济结构调整优化。持续推动钢铁、煤炭、电力、水泥等行业化解过剩产能，加大“僵尸企业”处置力度，促进落后产能退出。加快推进商品房去库存工作，推动房地产业健康发展。着力解决企业融资成本过高问题，积极实施贷款贴息等政策，切实降低企业杠杆率。加强对政府债务风险和金融风险的防控，坚决打好防范化解重大风险攻坚战。加大减税、降费、降低要素成本力度，进一步减轻企业负担。立足增强发展后劲，改善群众生活，着力补齐基础设施、公共服务、生态保护等方面短板。

（二）深化改革激发活力。扎实推进国资国企、财税金融、价格机制、投融资体制等重点领域改革。建立健全与供给侧结构性改革相适应的产业、土地、金融、财税、环保、价格等政策体系和长效机制。进一步放宽非公有制经济市场准入，推进建立公平竞争审查制度，努力消除各种隐性壁垒，营造公平竞争的市场环境。以混合所有制改革为突破口，积极推动央企属地注册，支持央企参与地方国企整合重组。推进投融资领域改革，大力推行政府和社会资本合作模式，做大用好各类投资基金，运用市场机制筹集城市建设资金，形成政府主导、市场运作、社会参与的多元投资格局。深化商事制度改革，推动更深层次的“多证合一”登记制度改革，进一步推进证照分离，打造具有国际竞争力的营商环境。加快推进以“放管服”为重点的行政审批制度改革，规范优化政务服务事项，提高行政审批效率，推动经济工作的着力点由“抓项目”向“抓环境”转变。进一步深化兵地融合，加强兵地人才交流，推动兵地协同发展。

（三）积极构建“五大中心”对外开放平台。聚焦“五通”，进一步完善“五大中心”规划政策体系，抓紧制定“五大中心”7项建设方案，积极争取将一批项目列入国家“一带一路”重大项目储备库。强化交通枢纽中心聚散功能，优化提升乌鲁木齐新客站服务功能，加快推进乌鲁木齐国际机场改扩建工程，充分发挥轨道交通高效便捷优势，构建现代综合立体交通体系。强化商贸物流中心交换功能，以加快建设国际陆港区为突破口，统筹推进综合保税区、空港物流园、中欧班列集结中心、城南经贸合作区等项目建设，大力促进投资和贸易自由化、便利化，以货物流带动人流、资金流，实现集货、建园、发展产业，构建现代物流全新模式。强化金融服务中心对外开放服务功能，积极推进人民币对周边国家货币直接挂牌和交易，不断拓宽跨境人民币结算业务范围，利用黄金市场功能，推动丝绸之路经济带沿线城市黄金实物循环，切实提升金融服务核心区建设的支撑保障能力。强化科教文化中心交流功能，积极推进丝绸之路经济带创新驱动发展试验区建设，加快建设中国—中亚科技合作中心、国家技术转移东部中心新疆分中心。围绕先进装备制造、高新技术产业、现代服务业等领域，重点建设一批示范带动力强、具备区域辐射能力的国际产业合作示范基地。积极争取新疆艺术学院丝绸之路艺术交流中心、中国新疆国际青年交流学院等项目落地建设。强化医疗服务中心合作功能，深化跨境远程医疗服务平台、跨境医疗旅游推介平台应用，建成乌鲁木齐国际医院、友爱医院，打造具有国际服务水平的重点医疗机构。

八、大力发展社会主义先进文化

“文化自信是一个国家、一个民族发展中更基本、更深沉、更持久的力量。”党的十九大从关系国家命运、关系民族发展的战略高度，向全党、全国人民发出了“坚定文化自信，推动社会主义文化繁荣兴盛”的号令。我们要把中国特色社会主义文化作为激励首府各族人民奋勇前进的强大精神力量，用中国特色社会主义文化引领人、教育人、塑造人，筑牢首府各族人民精神家园。

（一）大力弘扬社会主义核心价值观。坚持物质文明建设和精神文明建设“两手抓、两手都要硬”的战略方针，加强社会主义精神文明建设，为实现总目标提供有力的思想引领、精神支撑、智力支持。积极培育和践行社会主义核心价值观，加强理想信念教育，不忘本来、吸收外来、面向未来，把社会主义核心价值观融入社会发展方方面面，转化为人们的情感认同和行为习惯。持续深入地开展党的十九大精神和习近平新时代中国特色社会主义思想的学习宣传教育，强化“四个意识”，坚定“四个自信”，在全社会树牢中国特色社会主义理想信念。

（二）努力建设更高水平的文明城市。以成功创建全国文明城市为新起点，不断巩固全国文明城市创建成果，把党的十九大精神和以习近平同志为核心的党中央治疆方略融入文明城市建设全过程。加强社会公德、职业道德、家庭美德、个人品德建设，大力弘扬道德新风尚，提高各领域各行业文明服务水平，营造诚实守信的良好社会氛围。引导各族群众牢固树立马克思主义“五观”，坚定共产主义远大理想和中国特色社会主义共同理想，激励各族干部群众为实现党的十九大确定的目标任务而不懈奋斗。

（三）促进文化事业和文化产业蓬勃发展。围绕打造城市文化品牌，提升文艺原创力，不断推出文艺精品，增强首府文化艺术吸引力。进一步完善现代文化管理机制，稳步推进全市文化体制改革。鼓励和引导社会资本投资文化产业，积极创新文化生产经营机制，加快建设水磨沟区文化产业园，培育一批文化产业示范基地、文化产业示范园区和特色街区，壮大文化产业新业态。推广文化消费新模式，落实“互联网+”和“文化+”，搭建文化消费信息公共服务平台，构建线上、线下相结合的文化消费服务体系，培育文化消费新热点。

九、着力推进政府职能转变，建设清廉高效政府

我们要牢记使命，敢于担当，不断加强政府自身建设，全面提升依法履职能力和水平，努力建设人民满意的服务型政府。

（一）坚持党的领导。永远听党话、跟党走，在党的领导下认真履行政府职责，牢固树立政治意识、大局意识、核心意识和看齐意识，在政治立场、政治方向、政治原则、政治道路上同以习近平同志为核心的党中央保持高度一致，坚定信仰、忠诚践行习近平新时代中国特色社会主义思想，毫不动摇、百折不挠、不折不扣落实党中央、自治区党委和市委的决策部署。

（二）坚持依法行政。深入贯彻全面依法治市要求，尊崇法治、敬畏法律、依法行政，建设法治政府，促进首府治理体系和治理能力现代化。严格落实普法责任制，筑牢普法工作基石。依法接受人大及其常委会的监督，自觉接受人民政协的民主监督，认真听取人大代表、政协委员、民主党派、无党派人士和各人民团体意见。畅通问政渠道，主动接受社会舆论和群众监督，保障群众知情权、参与权和监督权。建设符合首府发展需求的高水平智库，落实重大问题集体决策、专家咨询、社会公示和听证制度，提高政府决策的科学化、民主化、法治化水平。

（三）坚持清正廉洁。巩固“三严三实”专题教育和“两学一做”学习教育成果，深入开展“不忘初心、牢记使命”主题教育。坚持不懈抓好中央八项规定和自治区党委、市委十条规定落实，驰而不息纠正“四风”“四气”，坚决查处“两 面 人”“两面派”。加强执法监察、效能监察和审计监督，加大行政问责力度，强化对行政权力的制约和监督，坚决纠正侵害群众利益的不正之风，积极营造风清气正的政治生态。

（四）坚持真抓实干。把履职尽责、落实见效贯穿工作始终，牢记“作风不实是我们最大的敌人”，真抓实干，敢于开拓，勇于担当，坚持把工作的着力点放在研究解决影响发展的重大问题上、放在解决群众关注的热点难点问题上。坚决整治行政不作为、乱作为、慢作为等问题，确保各项工作任务落实到位。严格监督考核，加强激励约束，强化失职追责，推动各级干部全面履职，担当尽责。建立容错纠错免责机制，为敢于担当、踏实做事、不谋私利的干部撑腰鼓劲，激发广大干部干事创业的积极性。

各位代表，党的十九大绘就的美好蓝图激励着我们，新时代的神圣使命召唤着我们，让我们更加紧密地团结在以习近平同志为核心的党中央周围，坚持以习近平新时代中国特色社会主义思想为指引，在自治区党委、自治区人民政府和市委的坚强领导下，把握新机遇、迎接新挑战、增创新优势、开创新局面，为建设团结和谐、繁荣富裕、文明进步、安居乐业的首府，在新时代谱写好中华民族伟大复兴中国梦的乌鲁木齐篇章而努力奋斗！
